# A linear memory algorithm for Baum-Welch training

**DOI:** 10.1186/1471-2105-6-231

**Published:** 2005-09-19

**Authors:** István Miklós, Irmtraud M Meyer

**Affiliations:** 1 MTA-ELTE Theoretical Biology and Ecology Group, Pázmány Péter sétány 1/c 1117 Budapest, Hungary.; 2 European Bioinformatics Institute, Wellcome Trust Genome Campus, Cambridge CB10 1SD, UK.

## Abstract

**Background:**

Baum-Welch training is an expectation-maximisation algorithm for training the emission and transition probabilities of hidden Markov models in a fully automated way. It can be employed as long as a training set of annotated sequences is known, and provides a rigorous way to derive parameter values which are guaranteed to be at least locally optimal. For complex hidden Markov models such as pair hidden Markov models and very long training sequences, even the most efficient algorithms for Baum-Welch training are currently too memory-consuming. This has so far effectively prevented the automatic parameter training of hidden Markov models that are currently used for biological sequence analyses.

**Results:**

We introduce the first linear space algorithm for Baum-Welch training. For a hidden Markov model with *M *states, *T *free transition and *E *free emission parameters, and an input sequence of length *L*, our new algorithm requires *O*(*M*) memory and *O*(*LMT*_*max *_(*T + E*)) time for one Baum-Welch iteration, where *T*_*max *_is the maximum number of states that any state is connected to. The most memory efficient algorithm until now was the checkpointing algorithm with *O*(log(*L*)*M*) memory and *O*(log(*L*)*LMT*_*max*_) time requirement. Our novel algorithm thus renders the memory requirement completely independent of the length of the training sequences. More generally, for an n-hidden Markov model and n input sequences of length *L*, the memory requirement of *O*(log(*L*)*L*^*n*-1 ^*M*) is reduced to *O*(*L*^*n*-1 ^*M*) memory while the running time is changed from *O*(log(*L*)*L*^*n *^*MT*_*max *_+ *L*^*n*^(*T *+ *E*)) to *O*(*L*^*n *^*MT*_*max *_(*T *+ *E*)).

An added advantage of our new algorithm is that a reduced time requirement can be traded for an increased memory requirement and *vice versa*, such that for any *c *∈ {1, ..., (*T *+ *E*)}, a time requirement of *L*^*n *^*MT*_*max *_*c *incurs a memory requirement of *L*^*n*-1 ^*M*(*T *+ *E *- *c*).

**Conclusion:**

For the large class of hidden Markov models used for example in gene prediction, whose number of states does not scale with the length of the input sequence, our novel algorithm can thus be both faster and more memory-efficient than any of the existing algorithms.

## Background

Hidden Markov Models (HMMs) are widely used in Bioinformatics [1], for example, in protein sequence alignment, protein family annotation [2,3] and gene-finding [4,5].

When an HMM consisting of *M *states is used to annotate an input sequence, its predictions crucially depend on its set of emission probabilities *ε *and transition probabilities . This is for example the case for the state path with the highest overall probability, the so-called optimal state path or Viterbi path [6], which is often reported as the predicted annotation of the input sequence.

When a new HMM is designed, it is usually quite easy to define its states and the transitions between them as these typically closely reflect the underlying problem. However, it can be quite difficult to assign values to its emission probabilities *ε *and transition probabilities . Ideally, they should be set up such that the model's predictions would perfectly reproduce the known annotation of a large and diverse set of input sequences.

The question is thus how to derive the best set of transition and emission probabilities from a given training set of annotated sequences. Two main scenarios have to be distinguished [1]:

(1) If we know the optimal state paths that correspond to the known annotation of the training sequences, the transition and emission probabilities can simply be set to the respective count frequencies within these optimal state paths, i.e. to their maximum likelihood estimators. If the training set is small or not diverse enough, pseudo-counts have to be added to avoid over-fitting.

(2) If we do not know the optimal state paths of the training sequences, either because their annotation is unknown or because their annotation does not unambiguously define a state path in the HMM, we can employ an expectation maximisation (EM) algorithm [7] such as the Baum-Welch algorithm [8] to derive the emission and transition probabilities in an iterative procedure which increases the overall log likelihood of the model in each iteration and which is guaranteed to converge at least to a local maximum. As in case (1), pseudo-counts or Dirichlet priors can be added to avoid over-fitting when the training set is small or not diverse enough.

## Methods and results

### Baum-Welch training

The Baum-Welch algorithm defines an iterative procedure in which the emission and transition probabilities in iteration *n *+ 1 are set to the number of times each transition and emission is *expected *to be used when analysing the training sequences with the set of emission and transition probabilities derived in the previous iteration *n*.

Let Ti,jn
 MathType@MTEF@5@5@+=feaafeart1ev1aaatCvAUfKttLearuWrP9MDH5MBPbIqV92AaeXatLxBI9gBaebbnrfifHhDYfgasaacH8akY=wiFfYdH8Gipec8Eeeu0xXdbba9frFj0=OqFfea0dXdd9vqai=hGuQ8kuc9pgc9s8qqaq=dirpe0xb9q8qiLsFr0=vr0=vr0dc8meaabaqaciaacaGaaeqabaqabeGadaaakeaacqWGubavdaqhaaWcbaGaemyAaKMaeiilaWIaemOAaOgabaGaemOBa4gaaaaa@3306@ denote the transition probability for going from state *i *to state *j *in iteration *n*, Ein(y)
 MathType@MTEF@5@5@+=feaafeart1ev1aaatCvAUfKttLearuWrP9MDH5MBPbIqV92AaeXatLxBI9gBaebbnrfifHhDYfgasaacH8akY=wiFfYdH8Gipec8Eeeu0xXdbba9frFj0=OqFfea0dXdd9vqai=hGuQ8kuc9pgc9s8qqaq=dirpe0xb9q8qiLsFr0=vr0=vr0dc8meaabaqaciaacaGaaeqabaqabeGadaaakeaacqWGfbqrdaqhaaWcbaGaemyAaKgabaGaemOBa4gaaOGaeiikaGIaemyEaKNaeiykaKcaaa@33E2@ the emission probability for emitting letter *y *in state *i *in iteration *n*, *P*(*X*) the probability of sequence *X*, and *x*_*k *_the *k*th letter in input sequence *X *which has length *L*. We also define *X*_*k *_as the sequence of letters from the beginning of sequence *X *up to sequence position *k*, (*x*_1_, ...*x*_*k*_). *X*^*k *^is defined as the sequence of letters from sequence position *k *+ 1 to the end of the sequence, (*x*_*k*+1_, ...*x*_*L*_).

For a given set of training sequences, *S*, the expectation maximisation update for transition probability Ti,jn
 MathType@MTEF@5@5@+=feaafeart1ev1aaatCvAUfKttLearuWrP9MDH5MBPbIqV92AaeXatLxBI9gBaebbnrfifHhDYfgasaacH8akY=wiFfYdH8Gipec8Eeeu0xXdbba9frFj0=OqFfea0dXdd9vqai=hGuQ8kuc9pgc9s8qqaq=dirpe0xb9q8qiLsFr0=vr0=vr0dc8meaabaqaciaacaGaaeqabaqabeGadaaakeaacqWGubavdaqhaaWcbaGaemyAaKMaeiilaWIaemOAaOgabaGaemOBa4gaaaaa@3306@, Ti,jn+1
 MathType@MTEF@5@5@+=feaafeart1ev1aaatCvAUfKttLearuWrP9MDH5MBPbIqV92AaeXatLxBI9gBaebbnrfifHhDYfgasaacH8akY=wiFfYdH8Gipec8Eeeu0xXdbba9frFj0=OqFfea0dXdd9vqai=hGuQ8kuc9pgc9s8qqaq=dirpe0xb9q8qiLsFr0=vr0=vr0dc8meaabaqaciaacaGaaeqabaqabeGadaaakeaacqWGubavdaqhaaWcbaGaemyAaKMaeiilaWIaemOAaOgabaGaemOBa4Maey4kaSIaeGymaedaaaaa@34D8@ can then be written as

Ti,jn+1=∑X∈Sti,jn(X)/P(X)∑j′∑X∈Sti,jn,(X)/P(X)where    ti,jn(X):=∑k=1Lfn(Xk,i)Ti,jnEjn(xk+1)bn(Xk+1,j)     (1)
 MathType@MTEF@5@5@+=feaafeart1ev1aaatCvAUfKttLearuWrP9MDH5MBPbIqV92AaeXatLxBI9gBaebbnrfifHhDYfgasaacH8akY=wiFfYdH8Gipec8Eeeu0xXdbba9frFj0=OqFfea0dXdd9vqai=hGuQ8kuc9pgc9s8qqaq=dirpe0xb9q8qiLsFr0=vr0=vr0dc8meaabaqaciaacaGaaeqabaqabeGadaaakeaafaqaaeGabaaabaGaemivaq1aa0baaSqaaiabdMgaPjabcYcaSiabdQgaQbqaaiabd6gaUjabgUcaRiabigdaXaaakiabg2da9maalaaabaaccaGae8xeIu+aaSbaaSqaaiabdIfayjabgIGiolabdofatbqabaGccqWG0baDdaqhaaWcbaGaemyAaKMaeiilaWIaemOAaOgabaGaemOBa4gaaOGaeiikaGIaemiwaGLaeiykaKIaei4la8IaemiuaaLaeiikaGIaemiwaGLaeiykaKcabaGae8xeIu+aaSbaaSqaaiqbdQgaQzaafaaabeaakiab=fHiLpaaBaaaleaacqWGybawcqGHiiIZcqWGtbWuaeqaaOGaemiDaq3aa0baaSqaaiabdMgaPjabcYcaSiabdQgaQbqaaiabd6gaUbaakiabcYcaSiabcIcaOiabdIfayjabcMcaPiabc+caViabdcfaqjabcIcaOiabdIfayjabcMcaPaaaaeGabaa5diaaxMaacqqG3bWDcqqGObaAcqqGLbqzcqqGYbGCcqqGLbqzcaaMc8UaaGPaVlaaykW7cqWG0baDdaqhaaWcbaGaemyAaKMaeiilaWIaemOAaOgabaGaemOBa4gaaOGaeiikaGIaemiwaGLaeiykaKIaeiOoaOJaeyypa0ZaaabCaeaacqWGMbGzdaahaaWcbeqaaiabd6gaUbaakiabcIcaOiabdIfaynaaBaaaleaacqWGRbWAcqGGSaalaeqaaOGaemyAaKMaeiykaKIaemivaq1aa0baaSqaaiabdMgaPjabcYcaSiabdQgaQbqaaiabd6gaUbaakiabdweafnaaDaaaleaacqWGQbGAaeaacqWGUbGBaaGccqGGOaakcqWG4baEdaWgaaWcbaGaem4AaSMaey4kaSIaeGymaedabeaakiabcMcaPaWcbaGaem4AaSMaeyypa0JaeGymaedabaGaemitaWeaniabggHiLdGccqWGIbGydaahaaWcbeqaaiabd6gaUbaakiabcIcaOiabdIfaynaaCaaaleqabaGaem4AaSMaey4kaSIaeGymaedaaOGaeiilaWIaemOAaOMaeiykaKcaaiaaxMaacaWLjaGaeiikaGIaeGymaeJaeiykaKcaaa@AC24@

The superfix *n *on the quantities on the right hand side indicates that they are based on the transition probabilities Ti,jn
 MathType@MTEF@5@5@+=feaafeart1ev1aaatCvAUfKttLearuWrP9MDH5MBPbIqV92AaeXatLxBI9gBaebbnrfifHhDYfgasaacH8akY=wiFfYdH8Gipec8Eeeu0xXdbba9frFj0=OqFfea0dXdd9vqai=hGuQ8kuc9pgc9s8qqaq=dirpe0xb9q8qiLsFr0=vr0=vr0dc8meaabaqaciaacaGaaeqabaqabeGadaaakeaacqWGubavdaqhaaWcbaGaemyAaKMaeiilaWIaemOAaOgabaGaemOBa4gaaaaa@3306@ and emission probabilities Ein(xk+1)
 MathType@MTEF@5@5@+=feaafeart1ev1aaatCvAUfKttLearuWrP9MDH5MBPbIqV92AaeXatLxBI9gBaebbnrfifHhDYfgasaacH8akY=wiFfYdH8Gipec8Eeeu0xXdbba9frFj0=OqFfea0dXdd9vqai=hGuQ8kuc9pgc9s8qqaq=dirpe0xb9q8qiLsFr0=vr0=vr0dc8meaabaqaciaacaGaaeqabaqabeGadaaakeaacqWGfbqrdaqhaaWcbaGaemyAaKgabaGaemOBa4gaaOGaeiikaGIaemiEaG3aaSbaaSqaaiabdUgaRjabgUcaRiabigdaXaqabaGccqGGPaqkaaa@3747@ of iteration *n*. *f*(*X*_*k*_, *i*): = *P*(*x*_1_, ...*x*_*k*_, *s*(*x*_*k*_) = *i*) is the so-called forward probability of the sequence up to and including sequence position *k*, requiring that sequence letter *x*_*k *_is read by state *i*. It is equal to the sum of probabilities of all state paths that finish in state *i *at sequence position *k*. The probability of sequence *X*, *P*(*X*), is therefore equal to *f*(*X*_*L*_, *End*). *b*(*X*^*k*^, *i*): = *P*(*x*_*k*+1_, ...*x*_*L*_|*s*(*x*_*k*_) = *i*) is the so-called backward probability of the sequence from sequence position *k *+ 1 to the end, given that the letter at sequence position *k*, *x*_*k*_, is read by state *i*. It is equal to the sum of probabilities of all state paths that start in state *i *at sequence position *k*.

For a given set of training sequences, *S*, the expectation maximisation update for emission probability Ein(y)
 MathType@MTEF@5@5@+=feaafeart1ev1aaatCvAUfKttLearuWrP9MDH5MBPbIqV92AaeXatLxBI9gBaebbnrfifHhDYfgasaacH8akY=wiFfYdH8Gipec8Eeeu0xXdbba9frFj0=OqFfea0dXdd9vqai=hGuQ8kuc9pgc9s8qqaq=dirpe0xb9q8qiLsFr0=vr0=vr0dc8meaabaqaciaacaGaaeqabaqabeGadaaakeaacqWGfbqrdaqhaaWcbaGaemyAaKgabaGaemOBa4gaaOGaeiikaGIaemyEaKNaeiykaKcaaa@33E2@, Ein+1(y)
 MathType@MTEF@5@5@+=feaafeart1ev1aaatCvAUfKttLearuWrP9MDH5MBPbIqV92AaeXatLxBI9gBaebbnrfifHhDYfgasaacH8akY=wiFfYdH8Gipec8Eeeu0xXdbba9frFj0=OqFfea0dXdd9vqai=hGuQ8kuc9pgc9s8qqaq=dirpe0xb9q8qiLsFr0=vr0=vr0dc8meaabaqaciaacaGaaeqabaqabeGadaaakeaacqWGfbqrdaqhaaWcbaGaemyAaKgabaGaemOBa4Maey4kaSIaeGymaedaaOGaeiikaGIaemyEaKNaeiykaKcaaa@35B4@, is

Ein+1(y)=∑X∈Sein(y,X)/P(X)∑y'∑X∈Sein(y',X)/P(X)where    ein(y,X):=∑k=1Lδxk,yfn(Xk,i)bn(Xk,i)     (2)
 MathType@MTEF@5@5@+=feaafeart1ev1aaatCvAUfKttLearuWrP9MDH5MBPbIqV92AaeXatLxBI9gBaebbnrfifHhDYfgasaacH8akY=wiFfYdH8Gipec8Eeeu0xXdbba9frFj0=OqFfea0dXdd9vqai=hGuQ8kuc9pgc9s8qqaq=dirpe0xb9q8qiLsFr0=vr0=vr0dc8meaabaqaciaacaGaaeqabaqabeGadaaakeaafaqaaeGabaaabaGaemyrau0aa0baaSqaaiabdMgaPbqaaiabd6gaUjabgUcaRiabigdaXaaakiabcIcaOiabdMha5jabcMcaPiabg2da9maalaaabaaccaGae8xeIu+aaSbaaSqaaiabdIfayjabgIGiolabdofatbqabaGccqWGLbqzdaqhaaWcbaGaemyAaKgabaGaemOBa4gaaOGaeiikaGIaemyEaKNaeiilaWIaemiwaGLaeiykaKIaei4la8IaemiuaaLaeiikaGIaemiwaGLaeiykaKcabaGae8xeIu+aaSbaaSqaaiabdMha5jabcEcaNaqabaGccqWFris5daWgaaWcbaGaemiwaGLaeyicI4Saem4uamfabeaakiabdwgaLnaaDaaaleaacqWGPbqAaeaacqWGUbGBaaGccqGGOaakcqWG5bqEcqGGNaWjcqGGSaalcqWGybawcqGGPaqkcqGGVaWlcqWGqbaucqGGOaakcqWGybawcqGGPaqkaaaabiqaaGsbcaWLjaGaee4DaCNaeeiAaGMaeeyzauMaeeOCaiNaeeyzauMaaGPaVlaaykW7caaMc8Uaemyzau2aa0baaSqaaiabdMgaPbqaaiabd6gaUbaakiabcIcaOiabdMha5jabcYcaSiabdIfayjabcMcaPiabcQda6iabg2da9maaqahabaGaeqiTdq2aaSbaaSqaaiabdIha4naaBaaameaacqWGRbWAaeqaaSGaeiilaWIaemyEaKhabeaakiabdAgaMnaaCaaaleqabaGaemOBa4gaaOGaeiikaGIaemiwaG1aaSbaaSqaaiabdUgaRjabcYcaSaqabaGccqWGPbqAcqGGPaqkaSqaaiabdUgaRjabg2da9iabigdaXaqaaiabdYeambqdcqGHris5aOGaemOyai2aaWbaaSqabeaacqWGUbGBaaGccqGGOaakcqWGybawdaahaaWcbeqaaiabdUgaRbaakiabcYcaSiabdMgaPjabcMcaPaaacaWLjaGaaCzcaiabcIcaOiabikdaYiabcMcaPaaa@A18B@

*δ *is the usual delta function with δxk,y
 MathType@MTEF@5@5@+=feaafeart1ev1aaatCvAUfKttLearuWrP9MDH5MBPbIqV92AaeXatLxBI9gBaebbnrfifHhDYfgasaacH8akY=wiFfYdH8Gipec8Eeeu0xXdbba9frFj0=OqFfea0dXdd9vqai=hGuQ8kuc9pgc9s8qqaq=dirpe0xb9q8qiLsFr0=vr0=vr0dc8meaabaqaciaacaGaaeqabaqabeGadaaakeaacqaH0oazdaWgaaWcbaGaemiEaG3aaSbaaWqaaiabdUgaRbqabaWccqGGSaalcqWG5bqEaeqaaaaa@33E7@ = 1 if *x*_*k *_= *y *and δxk,y
 MathType@MTEF@5@5@+=feaafeart1ev1aaatCvAUfKttLearuWrP9MDH5MBPbIqV92AaeXatLxBI9gBaebbnrfifHhDYfgasaacH8akY=wiFfYdH8Gipec8Eeeu0xXdbba9frFj0=OqFfea0dXdd9vqai=hGuQ8kuc9pgc9s8qqaq=dirpe0xb9q8qiLsFr0=vr0=vr0dc8meaabaqaciaacaGaaeqabaqabeGadaaakeaacqaH0oazdaWgaaWcbaGaemiEaG3aaSbaaWqaaiabdUgaRbqabaWccqGGSaalcqWG5bqEaeqaaaaa@33E7@ = 0 if *x*_*k *_≠ *y*. As before, the superfix *n *on the quantities on the right hand side indicates that they are calculated using the transition probabilities Ti,jn
 MathType@MTEF@5@5@+=feaafeart1ev1aaatCvAUfKttLearuWrP9MDH5MBPbIqV92AaeXatLxBI9gBaebbnrfifHhDYfgasaacH8akY=wiFfYdH8Gipec8Eeeu0xXdbba9frFj0=OqFfea0dXdd9vqai=hGuQ8kuc9pgc9s8qqaq=dirpe0xb9q8qiLsFr0=vr0=vr0dc8meaabaqaciaacaGaaeqabaqabeGadaaakeaacqWGubavdaqhaaWcbaGaemyAaKMaeiilaWIaemOAaOgabaGaemOBa4gaaaaa@3306@ and emission probabilities Ein(xk+1)
 MathType@MTEF@5@5@+=feaafeart1ev1aaatCvAUfKttLearuWrP9MDH5MBPbIqV92AaeXatLxBI9gBaebbnrfifHhDYfgasaacH8akY=wiFfYdH8Gipec8Eeeu0xXdbba9frFj0=OqFfea0dXdd9vqai=hGuQ8kuc9pgc9s8qqaq=dirpe0xb9q8qiLsFr0=vr0=vr0dc8meaabaqaciaacaGaaeqabaqabeGadaaakeaacqWGfbqrdaqhaaWcbaGaemyAaKgabaGaemOBa4gaaOGaeiikaGIaemiEaG3aaSbaaSqaaiabdUgaRjabgUcaRiabigdaXaqabaGccqGGPaqkaaa@3747@ of iteration *n*.

The forward and backward probabilities *f*^*n *^(*X*_*k*_, *i*) and *b*^*n*^(*X*^*k*^, *i*) can be calculated using the forward and backward algorithms [1] which are introduced in the following section.

#### Baum-Welch training using the forward and backward algorithm

The forward algorithm proposes a procedure for calculating the forward probabilities *f*(*X*_*k*_, *i*) in an iterative way. *f*(*X*_*k*_, *i*) is the sum of probabilities of all state paths that finish in state *i *at sequence position *k*.

The recursion starts with the initialisation

f(X0,i)={1   i=Start0   i≠Start
 MathType@MTEF@5@5@+=feaafeart1ev1aaatCvAUfKttLearuWrP9MDH5MBPbIqV92AaeXatLxBI9gBaebbnrfifHhDYfgasaacH8akY=wiFfYdH8Gipec8Eeeu0xXdbba9frFj0=OqFfea0dXdd9vqai=hGuQ8kuc9pgc9s8qqaq=dirpe0xb9q8qiLsFr0=vr0=vr0dc8meaabaqaciaacaGaaeqabaqabeGadaaakeaacqWGMbGzcqGGOaakcqWGybawdaWgaaWcbaGaeGimaadabeaakiabcYcaSiabdMgaPjabcMcaPiabg2da9maaceaabaqbaeqabiqaaaqaaiabigdaXiaaykW7caaMc8UaaGPaVlabdMgaPjabg2da9iabdofatjabdsha0jabdggaHjabdkhaYjabdsha0bqaaiabicdaWiaaykW7caaMc8UaaGPaVlabdMgaPjabgcMi5kabdofatjabdsha0jabdggaHjabdkhaYjabdsha0baaaiaawUhaaaaa@54AC@

where *Start *is the number of the start state in the HMM. The recursion proceeds towards higher sequence positions

f(Xk+1,i)=∑j=1Mf(Xk,j)Tj,iEi(xk+1)
 MathType@MTEF@5@5@+=feaafeart1ev1aaatCvAUfKttLearuWrP9MDH5MBPbIqV92AaeXatLxBI9gBaebbnrfifHhDYfgasaacH8akY=wiFfYdH8Gipec8Eeeu0xXdbba9frFj0=OqFfea0dXdd9vqai=hGuQ8kuc9pgc9s8qqaq=dirpe0xb9q8qiLsFr0=vr0=vr0dc8meaabaqaciaacaGaaeqabaqabeGadaaakeaacqWGMbGzcqGGOaakcqWGybawdaWgaaWcbaGaem4AaSMaey4kaSIaeGymaedabeaakiabcYcaSiabdMgaPjabcMcaPiabg2da9maaqahabaGaemOzaygaleaacqWGQbGAcqGH9aqpcqaIXaqmaeaacqWGnbqta0GaeyyeIuoakiabcIcaOiabdIfaynaaBaaaleaacqWGRbWAaeqaaOGaeiilaWIaemOAaOMaeiykaKIaemivaq1aaSbaaSqaaiabdQgaQjabcYcaSiabdMgaPbqabaGccqWGfbqrdaWgaaWcbaGaemyAaKgabeaakiabcIcaOiabdIha4naaBaaaleaacqWGRbWAcqGHRaWkcqaIXaqmaeqaaOGaeiykaKcaaa@549C@

and terminates with

P(X)=P(XL)=f(XL,End)=∑j=1Mf(XL,j)Tj,End
 MathType@MTEF@5@5@+=feaafeart1ev1aaatCvAUfKttLearuWrP9MDH5MBPbIqV92AaeXatLxBI9gBaebbnrfifHhDYfgasaacH8akY=wiFfYdH8Gipec8Eeeu0xXdbba9frFj0=OqFfea0dXdd9vqai=hGuQ8kuc9pgc9s8qqaq=dirpe0xb9q8qiLsFr0=vr0=vr0dc8meaabaqaciaacaGaaeqabaqabeGadaaakeaacqWGqbaucqGGOaakcqWGybawcqGGPaqkcqGH9aqpcqWGqbaucqGGOaakcqWGybawdaWgaaWcbaGaemitaWeabeaakiabcMcaPiabg2da9iabdAgaMjabcIcaOiabdIfaynaaBaaaleaacqWGmbataeqaaOGaeiilaWIaemyrauKaemOBa4MaemizaqMaeiykaKIaeyypa0ZaaabCaeaacqWGMbGzaSqaaiabdQgaQjabg2da9iabigdaXaqaaiabd2eanbqdcqGHris5aOGaeiikaGIaemiwaG1aaSbaaSqaaiabdYeambqabaGccqGGSaalcqWGQbGAcqGGPaqkcqWGubavdaWgaaWcbaGaemOAaOMaeiilaWIaemyrauKaemOBa4Maemizaqgabeaaaaa@5975@

where *End *is the number of the end state in the HMM. The recursion can be implemented as a dynamic programming procedure which works its way through a two-dimensional matrix, starting at the start of the sequence in the *Start *state and finishing at the end of the sequence in the *End *state of the HMM.

The backward algorithm calculates the backward probabilities *b*(*X*^*k*^, *i*) in a similar iterative way. *b*(*X*^*k*^, *i*) is the sum of probabilities of all state paths that start in state *i *at sequence position *k*. Opposed to the forward algorithm the backward algorithm starts at the end of the sequence in the *End *state and finishes at the start of the sequence in the *Start *state of the HMM.

The backward algorithm starts with the initialisation

b(XL,i)={1   i=End0   i≠End
 MathType@MTEF@5@5@+=feaafeart1ev1aaatCvAUfKttLearuWrP9MDH5MBPbIqV92AaeXatLxBI9gBaebbnrfifHhDYfgasaacH8akY=wiFfYdH8Gipec8Eeeu0xXdbba9frFj0=OqFfea0dXdd9vqai=hGuQ8kuc9pgc9s8qqaq=dirpe0xb9q8qiLsFr0=vr0=vr0dc8meaabaqaciaacaGaaeqabaqabeGadaaakeaacqWGIbGycqGGOaakcqWGybawdaahaaWcbeqaaiabdYeambaakiabcYcaSiabdMgaPjabcMcaPiabg2da9maaceaabaqbaeqabiqaaaqaaiabigdaXiaaykW7caaMc8UaaGPaVlabdMgaPjabg2da9iabdweafjabd6gaUjabdsgaKbqaaiabicdaWiaaykW7caaMc8UaaGPaVlabdMgaPjabgcMi5kabdweafjabd6gaUjabdsgaKbaaaiaawUhaaaaa@4ED8@

and continues towards lower sequence positions with the recursion

b(Xk,i)=∑j=1MEi(xk)Ti,jb(Xk+1,j)
 MathType@MTEF@5@5@+=feaafeart1ev1aaatCvAUfKttLearuWrP9MDH5MBPbIqV92AaeXatLxBI9gBaebbnrfifHhDYfgasaacH8akY=wiFfYdH8Gipec8Eeeu0xXdbba9frFj0=OqFfea0dXdd9vqai=hGuQ8kuc9pgc9s8qqaq=dirpe0xb9q8qiLsFr0=vr0=vr0dc8meaabaqaciaacaGaaeqabaqabeGadaaakeaacqWGIbGycqGGOaakcqWGybawdaahaaWcbeqaaiabdUgaRbaakiabcYcaSiabdMgaPjabcMcaPiabg2da9maaqahabaGaemyrau0aaSbaaSqaaiabdMgaPbqabaaabaGaemOAaOMaeyypa0JaeGymaedabaGaemyta0eaniabggHiLdGccqGGOaakcqWG4baEdaWgaaWcbaGaem4AaSgabeaakiabcMcaPiabdsfaunaaBaaaleaacqWGPbqAcqGGSaalcqWGQbGAaeqaaOGaemOyaiMaeiikaGIaemiwaG1aaWbaaSqabeaacqWGRbWAcqGHRaWkcqaIXaqmaaGccqGGSaalcqWGQbGAcqGGPaqkaaa@52A7@

and terminates with

P(X)=b(X1,Start)=∑j=1MTStart,jb(X1,j)
 MathType@MTEF@5@5@+=feaafeart1ev1aaatCvAUfKttLearuWrP9MDH5MBPbIqV92AaeXatLxBI9gBaebbnrfifHhDYfgasaacH8akY=wiFfYdH8Gipec8Eeeu0xXdbba9frFj0=OqFfea0dXdd9vqai=hGuQ8kuc9pgc9s8qqaq=dirpe0xb9q8qiLsFr0=vr0=vr0dc8meaabaqaciaacaGaaeqabaqabeGadaaakeaacqWGqbaucqGGOaakcqWGybawcqGGPaqkcqGH9aqpcqWGIbGycqGGOaakcqWGybawdaahaaWcbeqaaiabigdaXaaakiabcYcaSiabdofatjabdsha0jabdggaHjabdkhaYjabdsha0jabcMcaPiabg2da9maaqahabaGaemivaq1aaSbaaSqaaiabdofatjabdsha0jabdggaHjabdkhaYjabdsha0jabcYcaSiabdQgaQbqabaGccqWGIbGycqGGOaakcqWGybawdaahaaWcbeqaaiabigdaXaaakiabcYcaSiabdQgaQjabcMcaPaWcbaGaemOAaOMaeyypa0JaeGymaedabaGaemyta0eaniabggHiLdaaaa@5894@

As can be seen in the recursion steps of the forward and backward algorithms described above, the calculation of *f*(*X*_*k*+1_, *i*) requires at most *T*_*max *_previously calculated elements *f*(*X*_*k*_, *j*) for *j *∈ {1, ..*M*}. *T*_*max *_is the maximum number of states that any state of the model is connected to. Likewise, the calculation of *b*(*X*^*k*^, *i*) refers to at most *T*_*max *_elements *b*(*X*^*k*+*1*^, *j*) for *j *∈ {1, ..*M*}.

In order to continue the calculation of the forward and backward values *f*(*X*_*k*_, *i*) and *b*(*X*_*k*_, *i*) for all states *i *∈ {1, ..*M*} along the entire sequence, we thus only have to memorise *M *elements.

#### Baum-Welch training using the checkpointing algorithm

Unit now, the checkpointing algorithm [11-13] was the most efficient way to perform Baum-Welch training. The basic idea of the checkpointing algorithm is to perform the forward and backward algorithm by memorising the forward and backward values only in O(L)
 MathType@MTEF@5@5@+=feaafeart1ev1aaatCvAUfKttLearuWrP9MDH5MBPbIqV92AaeXatLxBI9gBaebbnrfifHhDYfgasaacH8akY=wiFfYdH8Gipec8Eeeu0xXdbba9frFj0=OqFfea0dXdd9vqai=hGuQ8kuc9pgc9s8qqaq=dirpe0xb9q8qiLsFr0=vr0=vr0dc8meaabaqaciaacaGaaeqabaqabeGadaaakeaacqWGpbWtcqGGOaakdaGcaaqaaiabdYeambWcbeaakiabcMcaPaaa@30CA@ columns along the sequence dimension of the dynamic programming table. The checkpointing algorithm starts with the forward algorithm, retaining only the forward values in O(L)
 MathType@MTEF@5@5@+=feaafeart1ev1aaatCvAUfKttLearuWrP9MDH5MBPbIqV92AaeXatLxBI9gBaebbnrfifHhDYfgasaacH8akY=wiFfYdH8Gipec8Eeeu0xXdbba9frFj0=OqFfea0dXdd9vqai=hGuQ8kuc9pgc9s8qqaq=dirpe0xb9q8qiLsFr0=vr0=vr0dc8meaabaqaciaacaGaaeqabaqabeGadaaakeaacqWGpbWtcqGGOaakdaGcaaqaaiabdYeambWcbeaakiabcMcaPaaa@30CA@ columns. These columns partition the dynamic programming table into O(L)
 MathType@MTEF@5@5@+=feaafeart1ev1aaatCvAUfKttLearuWrP9MDH5MBPbIqV92AaeXatLxBI9gBaebbnrfifHhDYfgasaacH8akY=wiFfYdH8Gipec8Eeeu0xXdbba9frFj0=OqFfea0dXdd9vqai=hGuQ8kuc9pgc9s8qqaq=dirpe0xb9q8qiLsFr0=vr0=vr0dc8meaabaqaciaacaGaaeqabaqabeGadaaakeaacqWGpbWtcqGGOaakdaGcaaqaaiabdYeambWcbeaakiabcMcaPaaa@30CA@ separate fields. The checkpointing algorithm then invokes the backward algorithm which memorises the backward values in a strip of length O(L)
 MathType@MTEF@5@5@+=feaafeart1ev1aaatCvAUfKttLearuWrP9MDH5MBPbIqV92AaeXatLxBI9gBaebbnrfifHhDYfgasaacH8akY=wiFfYdH8Gipec8Eeeu0xXdbba9frFj0=OqFfea0dXdd9vqai=hGuQ8kuc9pgc9s8qqaq=dirpe0xb9q8qiLsFr0=vr0=vr0dc8meaabaqaciaacaGaaeqabaqabeGadaaakeaacqWGpbWtcqGGOaakdaGcaaqaaiabdYeambWcbeaakiabcMcaPaaa@30CA@ as it moves along the sequence. When the backward calculation reaches the boundary of one field, the pre-calculated forward values of the neighbouring checkpointing column are used to calculate the corresponding forward values for that field. The forward and backward values of that field are then available at the same time and are used to calculate the corresponding values for the EM update.

The checkpointing algorithm can be further refined by using embedded checkpoints. With an embedding level of *k*, the forward values in O(L1k)
 MathType@MTEF@5@5@+=feaafeart1ev1aaatCvAUfKttLearuWrP9MDH5MBPbIqV92AaeXatLxBI9gBaebbnrfifHhDYfgasaacH8akY=wiFfYdH8Gipec8Eeeu0xXdbba9frFj0=OqFfea0dXdd9vqai=hGuQ8kuc9pgc9s8qqaq=dirpe0xb9q8qiLsFr0=vr0=vr0dc8meaabaqaciaacaGaaeqabaqabeGadaaakeaacqWGpbWtcqGGOaakcqWGmbatdaahaaWcbeqaamaaleaameaacqaIXaqmaeaacqWGRbWAaaaaaOGaeiykaKcaaa@3348@ columns of the initial calculation are memorised, thus defining O(L/L1k)=O(Lk−1k)
 MathType@MTEF@5@5@+=feaafeart1ev1aaatCvAUfKttLearuWrP9MDH5MBPbIqV92AaeXatLxBI9gBaebbnrfifHhDYfgasaacH8akY=wiFfYdH8Gipec8Eeeu0xXdbba9frFj0=OqFfea0dXdd9vqai=hGuQ8kuc9pgc9s8qqaq=dirpe0xb9q8qiLsFr0=vr0=vr0dc8meaabaqaciGacaGaaeqabaqabeGadaaakeaacqWGpbWtcqGGOaakcqWGmbatcqGGVaWlcqWGmbatdaahaaWcbeqaamaaleaameaacqaIXaqmaeaacqWGRbWAaaaaaOGaeiykaKIaeyypa0Jaem4ta8KaeiikaGIaemitaW0aaWbaaSqabeaadaWcbaadbaGaem4AaSMaeyOeI0IaeGymaedabaGaem4AaSgaaaaakiabcMcaPaaa@3F40@ long fields. When the memory-sparse calculation of the backward values reaches the field in question, the forward algorithm is invoked again to calculate the forward values for O(L1k)
 MathType@MTEF@5@5@+=feaafeart1ev1aaatCvAUfKttLearuWrP9MDH5MBPbIqV92AaeXatLxBI9gBaebbnrfifHhDYfgasaacH8akY=wiFfYdH8Gipec8Eeeu0xXdbba9frFj0=OqFfea0dXdd9vqai=hGuQ8kuc9pgc9s8qqaq=dirpe0xb9q8qiLsFr0=vr0=vr0dc8meaabaqaciaacaGaaeqabaqabeGadaaakeaacqWGpbWtcqGGOaakcqWGmbatdaahaaWcbeqaamaaleaameaacqaIXaqmaeaacqWGRbWAaaaaaOGaeiykaKcaaa@3348@ additional columns within that field. This procedure is iterated *k *times within the thus emerging fields. In the end, for each of the O(L1k)
 MathType@MTEF@5@5@+=feaafeart1ev1aaatCvAUfKttLearuWrP9MDH5MBPbIqV92AaeXatLxBI9gBaebbnrfifHhDYfgasaacH8akY=wiFfYdH8Gipec8Eeeu0xXdbba9frFj0=OqFfea0dXdd9vqai=hGuQ8kuc9pgc9s8qqaq=dirpe0xb9q8qiLsFr0=vr0=vr0dc8meaabaqaciaacaGaaeqabaqabeGadaaakeaacqWGpbWtcqGGOaakcqWGmbatdaahaaWcbeqaamaaleaameaacqaIXaqmaeaacqWGRbWAaaaaaOGaeiykaKcaaa@3348@-long k-sub-fields, the forward and backward values are simultaneously available and are used to calculate the corresponding values for the EM update. The time complexity of this algorithm for one Baum-Welch iteration and a given training sequence of length *L *is *O*(*kLMT*_*max *_+ *L*(*T *+ *E*)), since *k *forward and 1 backward algorithms have to be invoked, and the memory complexity is O(kL1kM)
 MathType@MTEF@5@5@+=feaafeart1ev1aaatCvAUfKttLearuWrP9MDH5MBPbIqV92AaeXatLxBI9gBaebbnrfifHhDYfgasaacH8akY=wiFfYdH8Gipec8Eeeu0xXdbba9frFj0=OqFfea0dXdd9vqai=hGuQ8kuc9pgc9s8qqaq=dirpe0xb9q8qiLsFr0=vr0=vr0dc8meaabaqaciaacaGaaeqabaqabeGadaaakeaacqWGpbWtcqGGOaakcqWGRbWAcqWGmbatdaahaaWcbeqaamaaleaameaacqaIXaqmaeaacqWGRbWAaaaaaOGaemyta0KaeiykaKcaaa@35CA@. For *k *= log(*L*), this amounts to a time requirement of *O*(log(*L*)*LMT*_*max *_+ *L*(*T *+ *E*)) and a memory requirement of *O*(log(*L*)*M*), since L1log⁡(L)
 MathType@MTEF@5@5@+=feaafeart1ev1aaatCvAUfKttLearuWrP9MDH5MBPbIqV92AaeXatLxBI9gBaebbnrfifHhDYfgasaacH8akY=wiFfYdH8Gipec8Eeeu0xXdbba9frFj0=OqFfea0dXdd9vqai=hGuQ8kuc9pgc9s8qqaq=dirpe0xb9q8qiLsFr0=vr0=vr0dc8meaabaqaciaacaGaaeqabaqabeGadaaakeaacqWGmbatdaahaaWcbeqaamaaleaameaacqaIXaqmaeaacyGGSbaBcqGGVbWBcqGGNbWzcqGGOaakcqWGmbatcqGGPaqkaaaaaaaa@35F7@ = *e*.

#### Baum-Welch training using the new algorithm

It is not trivial to see that the quantities Ti,jn+1
 MathType@MTEF@5@5@+=feaafeart1ev1aaatCvAUfKttLearuWrP9MDH5MBPbIqV92AaeXatLxBI9gBaebbnrfifHhDYfgasaacH8akY=wiFfYdH8Gipec8Eeeu0xXdbba9frFj0=OqFfea0dXdd9vqai=hGuQ8kuc9pgc9s8qqaq=dirpe0xb9q8qiLsFr0=vr0=vr0dc8meaabaqaciaacaGaaeqabaqabeGadaaakeaacqWGubavdaqhaaWcbaGaemyAaKMaeiilaWIaemOAaOgabaGaemOBa4Maey4kaSIaeGymaedaaaaa@34D8@ and Ein+1(y)
 MathType@MTEF@5@5@+=feaafeart1ev1aaatCvAUfKttLearuWrP9MDH5MBPbIqV92AaeXatLxBI9gBaebbnrfifHhDYfgasaacH8akY=wiFfYdH8Gipec8Eeeu0xXdbba9frFj0=OqFfea0dXdd9vqai=hGuQ8kuc9pgc9s8qqaq=dirpe0xb9q8qiLsFr0=vr0=vr0dc8meaabaqaciaacaGaaeqabaqabeGadaaakeaacqWGfbqrdaqhaaWcbaGaemyAaKgabaGaemOBa4Maey4kaSIaeGymaedaaOGaeiikaGIaemyEaKNaeiykaKcaaa@35B4@ of Equations 1 and 2 can be calculated in an even more memory-sparse way as both, the forward and the corresponding backward probabilities are needed at the same time in order to calculate the terms fn(Xk,i)Ti,jnEin(xk+1)bn(Xk+1,j)
 MathType@MTEF@5@5@+=feaafeart1ev1aaatCvAUfKttLearuWrP9MDH5MBPbIqV92AaeXatLxBI9gBaebbnrfifHhDYfgasaacH8akY=wiFfYdH8Gipec8Eeeu0xXdbba9frFj0=OqFfea0dXdd9vqai=hGuQ8kuc9pgc9s8qqaq=dirpe0xb9q8qiLsFr0=vr0=vr0dc8meaabaqaciaacaGaaeqabaqabeGadaaakeaacqWGMbGzdaahaaWcbeqaaiabd6gaUbaakiabcIcaOiabdIfaynaaBaaaleaacqWGRbWAaeqaaOGaeiilaWIaemyAaKMaeiykaKIaemivaq1aa0baaSqaaiabdMgaPjabcYcaSiabdQgaQbqaaiabd6gaUbaakiabdweafnaaDaaaleaacqWGPbqAaeaacqWGUbGBaaGccqGGOaakcqWG4baEdaWgaaWcbaGaem4AaSMaey4kaSIaeGymaedabeaakiabcMcaPiabdkgaInaaCaaaleqabaGaemOBa4gaaOGaeiikaGIaemiwaG1aaWbaaSqabeaacqWGRbWAcqGHRaWkcqaIXaqmaaGccqGGSaalcqWGQbGAcqGGPaqkaaa@52D1@ in ti,jn(X)
 MathType@MTEF@5@5@+=feaafeart1ev1aaatCvAUfKttLearuWrP9MDH5MBPbIqV92AaeXatLxBI9gBaebbnrfifHhDYfgasaacH8akY=wiFfYdH8Gipec8Eeeu0xXdbba9frFj0=OqFfea0dXdd9vqai=hGuQ8kuc9pgc9s8qqaq=dirpe0xb9q8qiLsFr0=vr0=vr0dc8meaabaqaciaacaGaaeqabaqabeGadaaakeaacqWG0baDdaqhaaWcbaGaemyAaKMaeiilaWIaemOAaOgabaGaemOBa4gaaOGaeiikaGIaemiwaGLaeiykaKcaaa@363B@ and δxk,yfn(Xk,i)bn(Xk,i)
 MathType@MTEF@5@5@+=feaafeart1ev1aaatCvAUfKttLearuWrP9MDH5MBPbIqV92AaeXatLxBI9gBaebbnrfifHhDYfgasaacH8akY=wiFfYdH8Gipec8Eeeu0xXdbba9frFj0=OqFfea0dXdd9vqai=hGuQ8kuc9pgc9s8qqaq=dirpe0xb9q8qiLsFr0=vr0=vr0dc8meaabaqaciaacaGaaeqabaqabeGadaaakeaacqaH0oazdaWgaaWcbaGaemiEaG3aaSbaaWqaaiabdUgaRbqabaWccqGGSaalcqWG5bqEaeqaaOGaemOzay2aaWbaaSqabeaacqWGUbGBaaGccqGGOaakcqWGybawdaWgaaWcbaGaem4AaSgabeaakiabcYcaSiabdMgaPjabcMcaPiabdkgaInaaCaaaleqabaGaemOBa4gaaOGaeiikaGIaemiwaG1aaWbaaSqabeaacqWGRbWAaaGccqGGSaalcqWGPbqAcqGGPaqkaaa@4742@ in ein(y,X)
 MathType@MTEF@5@5@+=feaafeart1ev1aaatCvAUfKttLearuWrP9MDH5MBPbIqV92AaeXatLxBI9gBaebbnrfifHhDYfgasaacH8akY=wiFfYdH8Gipec8Eeeu0xXdbba9frFj0=OqFfea0dXdd9vqai=hGuQ8kuc9pgc9s8qqaq=dirpe0xb9q8qiLsFr0=vr0=vr0dc8meaabaqaciaacaGaaeqabaqabeGadaaakeaacqWGLbqzdaqhaaWcbaGaemyAaKgabaGaemOBa4gaaOGaeiikaGIaemyEaKNaeiilaWIaemiwaGLaeiykaKcaaa@363B@ of Equations 1 and 2. A calculation of these quantities for each sequence position using a memory-sparse implementation (that would memorise only *M *values at a time) both for the forward and backward algorithm would require *L*-times more time, i.e. significantly more time. Also, an algorithm along the lines of the Hirschberg algorithm [9,10] cannot be applied as we cannot halve the dynamic programming table after the first recursion.

We here propose a new algorithm to calculate the quantities Ti,jn+1
 MathType@MTEF@5@5@+=feaafeart1ev1aaatCvAUfKttLearuWrP9MDH5MBPbIqV92AaeXatLxBI9gBaebbnrfifHhDYfgasaacH8akY=wiFfYdH8Gipec8Eeeu0xXdbba9frFj0=OqFfea0dXdd9vqai=hGuQ8kuc9pgc9s8qqaq=dirpe0xb9q8qiLsFr0=vr0=vr0dc8meaabaqaciaacaGaaeqabaqabeGadaaakeaacqWGubavdaqhaaWcbaGaemyAaKMaeiilaWIaemOAaOgabaGaemOBa4Maey4kaSIaeGymaedaaaaa@34D8@ and Ein+1(y)
 MathType@MTEF@5@5@+=feaafeart1ev1aaatCvAUfKttLearuWrP9MDH5MBPbIqV92AaeXatLxBI9gBaebbnrfifHhDYfgasaacH8akY=wiFfYdH8Gipec8Eeeu0xXdbba9frFj0=OqFfea0dXdd9vqai=hGuQ8kuc9pgc9s8qqaq=dirpe0xb9q8qiLsFr0=vr0=vr0dc8meaabaqaciaacaGaaeqabaqabeGadaaakeaacqWGfbqrdaqhaaWcbaGaemyAaKgabaGaemOBa4Maey4kaSIaeGymaedaaOGaeiikaGIaemyEaKNaeiykaKcaaa@35B4@ which are required for Baum-Welch training. Our algorithm requires *O*(*M*) memory and *O*(*LMT*_*max *_(*T *+ *E*)) time rather than *O*(log(*L*)*M*) memory and *O*(log(*L*{*LMT*_*max *_+ *L*(*T *+ *E*)) time.

The trick for coming up with a memory efficient algorithm is to realise that

• ti,jn(X)
 MathType@MTEF@5@5@+=feaafeart1ev1aaatCvAUfKttLearuWrP9MDH5MBPbIqV92AaeXatLxBI9gBaebbnrfifHhDYfgasaacH8akY=wiFfYdH8Gipec8Eeeu0xXdbba9frFj0=OqFfea0dXdd9vqai=hGuQ8kuc9pgc9s8qqaq=dirpe0xb9q8qiLsFr0=vr0=vr0dc8meaabaqaciaacaGaaeqabaqabeGadaaakeaacqWG0baDdaqhaaWcbaGaemyAaKMaeiilaWIaemOAaOgabaGaemOBa4gaaOGaeiikaGIaemiwaGLaeiykaKcaaa@363B@ and ein(y,X)
 MathType@MTEF@5@5@+=feaafeart1ev1aaatCvAUfKttLearuWrP9MDH5MBPbIqV92AaeXatLxBI9gBaebbnrfifHhDYfgasaacH8akY=wiFfYdH8Gipec8Eeeu0xXdbba9frFj0=OqFfea0dXdd9vqai=hGuQ8kuc9pgc9s8qqaq=dirpe0xb9q8qiLsFr0=vr0=vr0dc8meaabaqaciaacaGaaeqabaqabeGadaaakeaacqWGLbqzdaqhaaWcbaGaemyAaKgabaGaemOBa4gaaOGaeiikaGIaemyEaKNaeiilaWIaemiwaGLaeiykaKcaaa@363B@ in Equations 1 and 2 can be interpreted as a weighted sum of probabilities of state paths that satisfy certain constraints and that

• the weight of each state path is equal to the number of times that the constraint is fulfilled.

For example, ti,jn(X)
 MathType@MTEF@5@5@+=feaafeart1ev1aaatCvAUfKttLearuWrP9MDH5MBPbIqV92AaeXatLxBI9gBaebbnrfifHhDYfgasaacH8akY=wiFfYdH8Gipec8Eeeu0xXdbba9frFj0=OqFfea0dXdd9vqai=hGuQ8kuc9pgc9s8qqaq=dirpe0xb9q8qiLsFr0=vr0=vr0dc8meaabaqaciaacaGaaeqabaqabeGadaaakeaacqWG0baDdaqhaaWcbaGaemyAaKMaeiilaWIaemOAaOgabaGaemOBa4gaaOGaeiikaGIaemiwaGLaeiykaKcaaa@363B@ in the numerator in Equation 1 is the weighted sum of probabilities of state paths for sequence *X *that contain at least one *i *→ *j *transition, and the weight of each such state path in the sum is the number of times this transition occurs in the state path.

We now show how ti,jn(X)
 MathType@MTEF@5@5@+=feaafeart1ev1aaatCvAUfKttLearuWrP9MDH5MBPbIqV92AaeXatLxBI9gBaebbnrfifHhDYfgasaacH8akY=wiFfYdH8Gipec8Eeeu0xXdbba9frFj0=OqFfea0dXdd9vqai=hGuQ8kuc9pgc9s8qqaq=dirpe0xb9q8qiLsFr0=vr0=vr0dc8meaabaqaciaacaGaaeqabaqabeGadaaakeaacqWG0baDdaqhaaWcbaGaemyAaKMaeiilaWIaemOAaOgabaGaemOBa4gaaOGaeiikaGIaemiwaGLaeiykaKcaaa@363B@ in Equation 1 can be calculated in *O*(*M*) memory and *O*(*LMT*_*max*_) time. As the superfix *n *is only there to remind us that the calculation of ti,jn(X)
 MathType@MTEF@5@5@+=feaafeart1ev1aaatCvAUfKttLearuWrP9MDH5MBPbIqV92AaeXatLxBI9gBaebbnrfifHhDYfgasaacH8akY=wiFfYdH8Gipec8Eeeu0xXdbba9frFj0=OqFfea0dXdd9vqai=hGuQ8kuc9pgc9s8qqaq=dirpe0xb9q8qiLsFr0=vr0=vr0dc8meaabaqaciaacaGaaeqabaqabeGadaaakeaacqWG0baDdaqhaaWcbaGaemyAaKMaeiilaWIaemOAaOgabaGaemOBa4gaaOGaeiikaGIaemiwaGLaeiykaKcaaa@363B@ is based on the transition and emission probabilities of iteration *n *and as this index does not change in the calculation of ti,jn
 MathType@MTEF@5@5@+=feaafeart1ev1aaatCvAUfKttLearuWrP9MDH5MBPbIqV92AaeXatLxBI9gBaebbnrfifHhDYfgasaacH8akY=wiFfYdH8Gipec8Eeeu0xXdbba9frFj0=OqFfea0dXdd9vqai=hGuQ8kuc9pgc9s8qqaq=dirpe0xb9q8qiLsFr0=vr0=vr0dc8meaabaqaciaacaGaaeqabaqabeGadaaakeaacqWG0baDdaqhaaWcbaGaemyAaKMaeiilaWIaemOAaOgabaGaemOBa4gaaaaa@3346@, we discard it for simplicity sake in the following.

Let *t*_*i*, *j *_(*X*_*k*_, *l*) denote the weighted sum of probabilities of state paths that finish in state *l *at sequence position *k *of sequence *X *and that contain at least one *i *→ *j *transition, where the weight for each state path is equal to its number of *i *→ *j *transitions.

**Theorem 1: **The following algorithm calculates *t*_*i*, *j *_(*X*) in *O*(*M*) memory and *O*(*LMT*_*max*_) time. *t*_*i*, *j *_(*X*) is the weighted sum of probabilities of all state paths for sequence *X *that have at least one *i *→ *j *transition, where the weight for each state path is equal to its number of *i *→ *j *transitions.

The algorithm starts with the initialisation

f(X0,m)      ={1   m=Start0   m≠Startti,j(X0,m)=0
 MathType@MTEF@5@5@+=feaafeart1ev1aaatCvAUfKttLearuWrP9MDH5MBPbIqV92AaeXatLxBI9gBaebbnrfifHhDYfgasaacH8akY=wiFfYdH8Gipec8Eeeu0xXdbba9frFj0=OqFfea0dXdd9vqai=hGuQ8kuc9pgc9s8qqaq=dirpe0xb9q8qiLsFr0=vr0=vr0dc8meaabaqaciaacaGaaeqabaqabeGadaaakqaabeqaaiabdAgaMjabcIcaOiabdIfaynaaBaaaleaacqaIWaamaeqaaOGaeiilaWIaemyBa0MaeiykaKIaaGPaVlaaykW7caaMc8UaaGPaVlaaykW7caaMc8Uaeyypa0ZaaiqaaeaafaqabeGabaaabaGaeGymaeJaaGPaVlaaykW7caaMc8UaemyBa0Maeyypa0Jaem4uamLaemiDaqNaemyyaeMaemOCaiNaemiDaqhabaGaeGimaaJaaGPaVlaaykW7caaMc8UaemyBa0MaeyiyIKRaem4uamLaemiDaqNaemyyaeMaemOCaiNaemiDaqhaaaGaay5EaaaabaGaemiDaq3aaSbaaSqaaiabdMgaPjabcYcaSiabdQgaQbqabaGccqGGOaakcqWGybawdaWgaaWcbaGaeGimaadabeaakiabcYcaSiabd2gaTjabcMcaPiabg2da9iabicdaWaaaaa@6B92@

and proceeds via the following recursion

f(Xk+1,m)       =∑n=1Mf(Xk,n)Tn,mEm(xk+1)ti,j(Xk+1,m)={∑n=1Mti,j(Xk,n)Tn,mEm(xk+1)   m≠jf(Xk,i)Ti,mEm(xk+1)+                    m=j∑n=1Mti,j(Xk,n)Tn,mEm(xk+1)     (3)
 MathType@MTEF@5@5@+=feaafeart1ev1aaatCvAUfKttLearuWrP9MDH5MBPbIqV92AaeXatLxBI9gBaebbnrfifHhDYfgasaacH8akY=wiFfYdH8Gipec8Eeeu0xXdbba9frFj0=OqFfea0dXdd9vqai=hGuQ8kuc9pgc9s8qqaq=dirpe0xb9q8qiLsFr0=vr0=vr0dc8meaabaqaciaacaGaaeqabaqabeGadaaakqaaeeqaaiabdAgaMjabcIcaOiabdIfaynaaBaaaleaacqWGRbWAcqGHRaWkcqaIXaqmaeqaaOGaeiilaWIaemyBa0MaeiykaKIaaGPaVlaaykW7caaMc8UaaGPaVlaaykW7caaMc8UaaGPaVlabg2da9maaqahabaGaemOzayMaeiikaGIaemiwaG1aaSbaaSqaaiabdUgaRbqabaGccqGGSaalcqWGUbGBcqGGPaqkaSqaaiabd6gaUjabg2da9iabigdaXaqaaiabd2eanbqdcqGHris5aOGaemivaq1aaSbaaSqaaiabd6gaUjabcYcaSiabd2gaTbqabaGccqWGfbqrdaWgaaWcbaGaemyBa0gabeaakiabcIcaOiabdIha4naaBaaaleaacqWGRbWAcqGHRaWkcqaIXaqmaeqaaOGaeiykaKcabaGaemiDaq3aaSbaaSqaaiabdMgaPjabcYcaSiabdQgaQbqabaGccqGGOaakcqWGybawdaWgaaWcbaGaem4AaSMaey4kaSIaeGymaedabeaakiabcYcaSiabd2gaTjabcMcaPiabg2da9maaceaabaqbaeqabiqaaaqaamaaqadabaGaemiDaq3aaSbaaSqaaiabdMgaPjabcYcaSiabdQgaQbqabaGccqGGOaakcqWGybawdaWgaaWcbaGaem4AaSgabeaakiabcYcaSiabd6gaUjabcMcaPaWcbaGaemOBa4Maeyypa0JaeGymaedabaGaemyta0eaniabggHiLdGccqWGubavdaWgaaWcbaGaemOBa4MaeiilaWIaemyBa0gabeaakiabdweafnaaBaaaleaacqWGTbqBaeqaaOGaeiikaGIaemiEaG3aaSbaaSqaaiabdUgaRjabgUcaRiabigdaXaqabaGccqGGPaqkcaaMc8UaaGPaVlaaykW7cqWGTbqBcqGHGjsUcqWGQbGAaqaabeqaaaqaaiabdAgaMjabcIcaOiabdIfaynaaBaaaleaacqWGRbWAaeqaaOGaeiilaWIaemyAaKMaeiykaKIaemivaq1aaSbaaSqaaiabdMgaPjabcYcaSiabd2gaTbqabaGccqWGfbqrdaWgaaWcbaGaemyBa0gabeaakiabcIcaOiabdIha4naaBaaaleaacqWGRbWAcqGHRaWkcqaIXaqmaeqaaOGaeiykaKIaey4kaSIaaGPaVlaaykW7caaMc8UaaGPaVlaaykW7caaMc8UaaGPaVlaaykW7caaMc8UaaGPaVlaaykW7caaMc8UaaGPaVlaaykW7caaMc8UaaGPaVlaaykW7caaMc8UaaGPaVlaaykW7cqWGTbqBcqGH9aqpcqWGQbGAaeaadaaeWaqaaiabdsha0naaBaaaleaacqWGPbqAcqGGSaalcqWGQbGAaeqaaOGaeiikaGIaemiwaG1aaSbaaSqaaiabdUgaRbqabaGccqGGSaalcqWGUbGBcqGGPaqkaSqaaiabd6gaUjabg2da9iabigdaXaqaaiabd2eanbqdcqGHris5aOGaemivaq1aaSbaaSqaaiabd6gaUjabcYcaSiabd2gaTbqabaGccqWGfbqrdaWgaaWcbaGaemyBa0gabeaakiabcIcaOiabdIha4naaBaaaleaacqWGRbWAcqGHRaWkcqaIXaqmaeqaaOGaeiykaKcaaaaacaGL7baacaWLjaGaaCzcaiabcIcaOiabiodaZiabcMcaPaaaaa@F84B@

and finishes with

     P(X)=f(XL,End)   =∑n=1Mf(XL,n)Tn,Endti,j(X)=ti,j(XL,End)={∑n=1Mti,j(XL,n)Tn,Endf(XL,i)Ti,End+∑n=1Mti,End(Xk,n)Tn,EndEnd≠jEnd=j     (4)
 MathType@MTEF@5@5@+=feaafeart1ev1aaatCvAUfKttLearuWrP9MDH5MBPbIqV92AaeXatLxBI9gBaebbnrfifHhDYfgasaacH8akY=wiFfYdH8Gipec8Eeeu0xXdbba9frFj0=OqFfea0dXdd9vqai=hGuQ8kuc9pgc9s8qqaq=dirpe0xb9q8qiLsFr0=vr0=vr0dc8meaabaqaciaacaGaaeqabaqabeGadaaakqaabeqaauaabaqaceaaaeaacaaMc8UaaGPaVlaaykW7caaMc8UaaGPaVlabdcfaqjabcIcaOiabdIfayjabcMcaPiabg2da9iabdAgaMjabcIcaOiabdIfaynaaBaaaleaacqWGmbataeqaaOGaeiilaWIaemyrauKaemOBa4MaemizaqMaeiykaKIaaGPaVlaaykW7caaMc8Uaeyypa0ZaaabCaeaacqWGMbGzcqGGOaakcqWGybawdaWgaaWcbaGaemitaWeabeaakiabcYcaSiabd6gaUjabcMcaPaWcbaGaemOBa4Maeyypa0JaeGymaedabaGaemyta0eaniabggHiLdGccqWGubavdaWgaaWcbaGaemOBa4MaeiilaWIaemyrauKaemOBa4MaemizaqgabeaaaOqaaiabdsha0naaBaaaleaacqWGPbqAcqGGSaalcqWGQbGAaeqaaOGaeiikaGIaemiwaGLaeiykaKIaeyypa0JaemiDaq3aaSbaaSqaaiabdMgaPjabcYcaSiabdQgaQbqabaGccqGGOaakcqWGybawdaWgaaWcbaGaemitaWeabeaakiabcYcaSiabdweafjabd6gaUjabdsgaKjabcMcaPiabg2da9maaceaabaqbaeaabiqaaaabaeqabaWaaabmaeaacqWG0baDdaWgaaWcbaGaemyAaKMaeiilaWIaemOAaOgabeaakiabcIcaOiabdIfaynaaBaaaleaacqWGmbataeqaaOGaeiilaWIaemOBa4MaeiykaKcaleaacqWGUbGBcqGH9aqpcqaIXaqmaeaacqWGnbqta0GaeyyeIuoakiabdsfaunaaBaaaleaacqWGUbGBcqGGSaalcqWGfbqrcqWGUbGBcqWGKbazaeqaaaGcbaaaaqaabeqaaiabdAgaMjabcIcaOiabdIfaynaaBaaaleaacqWGmbataeqaaOGaeiilaWIaemyAaKMaeiykaKIaemivaq1aaSbaaSqaaiabdMgaPjabcYcaSiabdweafjabd6gaUjabdsgaKbqabaGccqGHRaWkaeaadaaeWaqaaiabdsha0naaBaaaleaacqWGPbqAcqGGSaalcqWGfbqrcqWGUbGBcqWGKbazaeqaaOGaeiikaGIaemiwaG1aaSbaaSqaaiabdUgaRbqabaGccqGGSaalcqWGUbGBcqGGPaqkaSqaaiabd6gaUjabg2da9iabigdaXaqaaiabd2eanbqdcqGHris5aOGaemivaq1aaSbaaSqaaiabd6gaUjabcYcaSiabdweafjabd6gaUjabdsgaKbqabaaaaaaakiaawUhaauaabeqaeeaaaaqaaiabdweafjabd6gaUjabdsgaKjabgcMi5kabdQgaQbqaaaqaaiabdweafjabd6gaUjabdsgaKjabg2da9iabdQgaQbqaaaaaaaGaaCzcaiaaxMaacqGGOaakcqaI0aancqGGPaqkaeaaaaaa@D08E@

Proof:

(1) It is obvious that the recursion requires only *O*(*M*) memory as the calculation of all *f*(*X*_*k*+1_, *m*) values with *m *∈ {1, ..*M*} requires only access to the *M *previous *f*(*X*_*k*_, *n*) values with *n *∈ {1, ..*M*}. Likewise, the calculations of all *t*_*i*, *j*_(*X*_*k*+1_, *m*) values with *m *∈ {1, ..*M*} refer only to *M *elements *t*_*i*, *j*_(*X*_*k*_, *n*) with *n *∈ {1, ..*M*}. We therefore have to remember only a thin "slice" of *t*_*i*, *j *_and *f *values at sequence position *k *in order to be able to calculate the *t*_*i*, *j *_and *f *values for the next sequence position *k *+ 1. The time requirement to calculate *t*_*i*, *j *_is *O*(*LMT*_*max*_): for every sequence position and for every state in the HMM, we have to sum at most *T*_*max *_terms in order to calculate the backward and forward terms.

(2) The *f*(*X*_*k*_, *m*) values are identical to the previously defined forward probabilities and are calculated in the same way as in the forward algorithm.

(3) We now prove by induction that *t*_*i*, *j*_(*X*_*k*_, *l*) is equal to the weighted sum of probabilities of state paths that have at least one *i *→ *j *transition and that finish at sequence position *k *in state *l*, the weight of each state path being equal to its number of *i *→ *j *transitions.

Initialisation step (sequence position *k = *0): *t*_*i*, *j*_(*X*_0_, *m*) = 0 is true as the sum of probabilities of state paths that finish in state *m *at sequence position 0 and that have at least one *i *→ *j *transition is zero. Induction step *k *→ *k *+ 1: We now show that if Equation 3 is true for sequence position *k*, it is also true for *k + *1. We have to distinguish two cases:

(i) case *m = j*:

ti,j(Xk+1,m)=f(Xk,i)Ti,jEj(xk+1)+     (5)
 MathType@MTEF@5@5@+=feaafeart1ev1aaatCvAUfKttLearuWrP9MDH5MBPbIqV92AaeXatLxBI9gBaebbnrfifHhDYfgasaacH8akY=wiFfYdH8Gipec8Eeeu0xXdbba9frFj0=OqFfea0dXdd9vqai=hGuQ8kuc9pgc9s8qqaq=dirpe0xb9q8qiLsFr0=vr0=vr0dc8meaabaqaciaacaGaaeqabaqabeGadaaakeaacqWG0baDdaWgaaWcbaGaemyAaKMaeiilaWIaemOAaOgabeaakiabcIcaOiabdIfaynaaBaaaleaacqWGRbWAcqGHRaWkcqaIXaqmaeqaaOGaeiilaWIaemyBa0MaeiykaKIaeyypa0JaemOzayMaeiikaGIaemiwaG1aaSbaaSqaaiabdUgaRbqabaGccqGGSaalcqWGPbqAcqGGPaqkcqWGubavdaWgaaWcbaGaemyAaKMaeiilaWIaemOAaOgabeaakiabdweafnaaBaaaleaacqWGQbGAaeqaaOGaeiikaGIaemiEaG3aaSbaaSqaaiabdUgaRjabgUcaRiabigdaXaqabaGccqGGPaqkcqGHRaWkcaWLjaGaaCzcaiabcIcaOiabiwda1iabcMcaPaaa@569C@

∑n=1Mti,j(Xk,n)Tn,jEj(Xk+1)     (6)
 MathType@MTEF@5@5@+=feaafeart1ev1aaatCvAUfKttLearuWrP9MDH5MBPbIqV92AaeXatLxBI9gBaebbnrfifHhDYfgasaacH8akY=wiFfYdH8Gipec8Eeeu0xXdbba9frFj0=OqFfea0dXdd9vqai=hGuQ8kuc9pgc9s8qqaq=dirpe0xb9q8qiLsFr0=vr0=vr0dc8meaabaqaciaacaGaaeqabaqabeGadaaakeaadaaeWbqaaiabdsha0naaBaaaleaacqWGPbqAcqGGSaalcqWGQbGAaeqaaOGaeiikaGIaemiwaG1aaSbaaSqaaiabdUgaRbqabaGccqGGSaalcqWGUbGBcqGGPaqkaSqaaiabd6gaUjabg2da9iabigdaXaqaaiabd2eanbqdcqGHris5aOGaemivaq1aaSbaaSqaaiabd6gaUjabcYcaSiabdQgaQbqabaGccqWGfbqrdaWgaaWcbaGaemOAaOgabeaakiabcIcaOiabdIfaynaaBaaaleaacqWGRbWAcqGHRaWkcqaIXaqmaeqaaOGaeiykaKIaaCzcaiaaxMaacqGGOaakcqaI2aGncqGGPaqkaaa@516A@

The first term, see right hand side of 5, is the sum of probabilities of state paths that finish at sequence position *k + *1 and whose last transition is from *i *→ *j*. The second term, see 6, is the sum of probabilities of state paths that finish at sequence position *k *+ 1 and that already have at least one *i *→ *j *transition. Note that the term in 6 also contains a contribution for *n = i*. This ensures that the weight of those state path that already have at least one *i *→ *j *transition is correctly increased by 1. The sum, *t*_*i*, *j*_(*X*_*k*+1_, *m*), is therefore the weighted sum of probabilities of state paths that finish in sequence position *k *+ 1 and contain at least one *i *→ *j *transition. Each state path's weight in the sum is equal to its number of *i *→ *j *transitions.

(ii) case *m *≠ *j*:

∑n=1Mti,j(Xk,n)Tn,jEj(Xk+1)     (6)
 MathType@MTEF@5@5@+=feaafeart1ev1aaatCvAUfKttLearuWrP9MDH5MBPbIqV92AaeXatLxBI9gBaebbnrfifHhDYfgasaacH8akY=wiFfYdH8Gipec8Eeeu0xXdbba9frFj0=OqFfea0dXdd9vqai=hGuQ8kuc9pgc9s8qqaq=dirpe0xb9q8qiLsFr0=vr0=vr0dc8meaabaqaciaacaGaaeqabaqabeGadaaakeaadaaeWbqaaiabdsha0naaBaaaleaacqWGPbqAcqGGSaalcqWGQbGAaeqaaOGaeiikaGIaemiwaG1aaSbaaSqaaiabdUgaRbqabaGccqGGSaalcqWGUbGBcqGGPaqkaSqaaiabd6gaUjabg2da9iabigdaXaqaaiabd2eanbqdcqGHris5aOGaemivaq1aaSbaaSqaaiabd6gaUjabcYcaSiabdQgaQbqabaGccqWGfbqrdaWgaaWcbaGaemOAaOgabeaakiabcIcaOiabdIfaynaaBaaaleaacqWGRbWAcqGHRaWkcqaIXaqmaeqaaOGaeiykaKIaaCzcaiaaxMaacqGGOaakcqaI2aGncqGGPaqkaaa@516A@

The expression on the right hand side is the weighted sum of probabilities of state paths that finish in sequence position *k *+ 1 and contain at least one *i *→ *j *transition.

We have therefore shown that if Equation 3 is true for sequence position *k*, it is also true for sequence position *k *+ 1. This concludes the proof of theorem 1.     □

It is easy to show that *e*_*i*_(*y*, *X*) in Equation 2 can also be calculated in *O*(*M*) memory and *O*(*LMT*_*max*_) time in a similar way as *t*_*i*, *j*_(*X*). Let *e*_*i*_(*y*, *X*_*k*_, *l*) denote the weighted sum of probabilities of state paths that finish at sequence position *k *in state *l *and for which state *i *reads letter *y *at least once, the weight of each state path being equal to the number of times state *i *reads letter *y*. As in the calculation of *t*_*i*, *j*_(*X*) we again omit the superfix *n *as the calculation of *e*_*i*_(*y*, *X*) is again entirely based on the transition and emission probabilities of iteration *n*.

**Theorem 2: ***e*_*i*_(*y*, *X*) can be calculated in *O*(*M*) memory and *O*(*LMT*_*max*_) time using the following algorithm. *e*_*i*_(*y*, *X*) is the weighted sum of probabilities of state paths for sequence *X *that read letter *y *in state *i *at least once, the weight of each state path being equal to the number of times letter *y *is read by state *i*.

Initialisation step:

f(X0,m)         ={1   m=Start0   m≠Startei(y,X0,m)=0
 MathType@MTEF@5@5@+=feaafeart1ev1aaatCvAUfKttLearuWrP9MDH5MBPbIqV92AaeXatLxBI9gBaebbnrfifHhDYfgasaacH8akY=wiFfYdH8Gipec8Eeeu0xXdbba9frFj0=OqFfea0dXdd9vqai=hGuQ8kuc9pgc9s8qqaq=dirpe0xb9q8qiLsFr0=vr0=vr0dc8meaabaqaciaacaGaaeqabaqabeGadaaakqaaeeqaaiabdAgaMjabcIcaOiabdIfaynaaBaaaleaacqaIWaamaeqaaOGaeiilaWIaemyBa0MaeiykaKIaaGPaVlaaykW7caaMc8UaaGPaVlaaykW7caaMc8UaaGPaVlaaykW7caaMc8Uaeyypa0ZaaiqaaeaafaqabeGabaaabaGaeGymaeJaaGPaVlaaykW7caaMc8UaemyBa0Maeyypa0Jaem4uamLaemiDaqNaemyyaeMaemOCaiNaemiDaqhabaGaeGimaaJaaGPaVlaaykW7caaMc8UaemyBa0MaeyiyIKRaem4uamLaemiDaqNaemyyaeMaemOCaiNaemiDaqhaaaGaay5EaaaabaGaemyzau2aaSbaaSqaaiabdMgaPbqabaGccqGGOaakcqWG5bqEcqGGSaalcqWGybawdaWgaaWcbaGaeGimaadabeaakiabcYcaSiabd2gaTjabcMcaPiabg2da9iabicdaWaaaaa@7036@

Recursion:

f(Xk+1,m)=∑n=1Mf(Xk,n)Tn,mEm(xk+1)ei(y,Xk+1,m)={∑n=1Mei(y,Xk,n)Tn,mEm(xk+1)if   m≠i   or   xk+1≠yf(Xk,i)Ti,mEm(xk+1)+∑n=1Mei(y,Xk,n)Tn,mEm(xk+1)if   m=i   and   xk+1=y
 MathType@MTEF@5@5@+=feaafeart1ev1aaatCvAUfKttLearuWrP9MDH5MBPbIqV92AaeXatLxBI9gBaebbnrfifHhDYfgasaacH8akY=wiFfYdH8Gipec8Eeeu0xXdbba9frFj0=OqFfea0dXdd9vqai=hGuQ8kuc9pgc9s8qqaq=dirpe0xb9q8qiLsFr0=vr0=vr0dc8meaabaqaciaacaGaaeqabaqabeGadaaakqaaeeqaaiabdAgaMjabcIcaOiabdIfaynaaBaaaleaacqWGRbWAcqGHRaWkcqaIXaqmaeqaaOGaeiilaWIaemyBa0MaeiykaKIaeyypa0ZaaabCaeaacqWGMbGzcqGGOaakcqWGybawdaWgaaWcbaGaem4AaSgabeaakiabcYcaSiabd6gaUjabcMcaPiabdsfaunaaBaaaleaacqWGUbGBcqGGSaalcqWGTbqBaeqaaOGaemyrau0aaSbaaSqaaiabd2gaTbqabaGccqGGOaakcqWG4baEdaWgaaWcbaGaem4AaSMaey4kaSIaeGymaedabeaakiabcMcaPaWcbaGaemOBa4Maeyypa0JaeGymaedabaGaemyta0eaniabggHiLdaakeaacqWGLbqzdaWgaaWcbaGaemyAaKgabeaakiabcIcaOiabdMha5jabcYcaSiabdIfaynaaBaaaleaacqWGRbWAcqGHRaWkcqaIXaqmaeqaaOGaeiilaWIaemyBa0MaeiykaKIaeyypa0tbaeqabeqaaaqaamaaceaaeaqabeaadaaeWaqaaiabdwgaLnaaBaaaleaacqWGPbqAaeqaaOGaeiikaGIaemyEaKNaeiilaWIaemiwaG1aaSbaaSqaaiabdUgaRbqabaGccqGGSaalcqWGUbGBcqGGPaqkcqWGubavdaWgaaWcbaGaemOBa4MaeiilaWIaemyBa0gabeaakiabdweafnaaBaaaleaacqWGTbqBaeqaaOGaeiikaGIaemiEaG3aaSbaaSqaaiabdUgaRjabgUcaRiabigdaXaqabaGccqGGPaqkaSqaaiabd6gaUjabg2da9iabigdaXaqaaiabd2eanbqdcqGHris5aaGcbaacbaGae8xAaKMae8NzayMaaGPaVlaaykW7caaMc8UaemyBa0MaeyiyIKRaemyAaKMaaGPaVlaaykW7caaMc8Uaee4Ba8MaeeOCaiNaaGPaVlaaykW7caaMc8UaemiEaG3aaSbaaSqaaiabdUgaRjabgUcaRiabigdaXaqabaGccqGHGjsUcqWG5bqEaeaaaeaacqWGMbGzcqGGOaakcqWGybawdaWgaaWcbaGaem4AaSgabeaakiabcYcaSiabdMgaPjabcMcaPiabdsfaunaaBaaaleaacqWGPbqAcqGGSaalcqWGTbqBaeqaaOGaemyrau0aaSbaaSqaaiabd2gaTbqabaGccqGGOaakcqWG4baEdaWgaaWcbaGaem4AaSMaey4kaSIaeGymaedabeaakiabcMcaPiabgUcaRaqaamaaqadabaGaemyzau2aaSbaaSqaaiabdMgaPbqabaGccqGGOaakcqWG5bqEcqGGSaalcqWGybawdaWgaaWcbaGaem4AaSgabeaakiabcYcaSiabd6gaUjabcMcaPiabdsfaunaaBaaaleaacqWGUbGBcqGGSaalcqWGTbqBaeqaaOGaemyrau0aaSbaaSqaaiabd2gaTbqabaGccqGGOaakcqWG4baEdaWgaaWcbaGaem4AaSMaey4kaSIaeGymaedabeaakiabcMcaPaWcbaGaemOBa4Maeyypa0JaeGymaedabaGaemyta0eaniabggHiLdaakeaacqWFPbqAcqWFMbGzcaaMc8UaaGPaVlaaykW7cqWGTbqBcqGH9aqpcqWGPbqAcaaMc8UaaGPaVlaaykW7cqWFHbqycqWFUbGBcqWFKbazcaaMc8UaaGPaVlaaykW7cqWG4baEdaWgaaWcbaGaem4AaSMaey4kaSIaeGymaedabeaakiabg2da9iabdMha5baacaGL7baaaaaaaaa@FD7B@

Termination step:

P(X)=f(XL,End)=∑n+1Mf(XL,n)Tn,End     (7)ei(y,X)=ei(y,XL,End)=∑n+1Mei(y,XL,n)Tn,End
 MathType@MTEF@5@5@+=feaafeart1ev1aaatCvAUfKttLearuWrP9MDH5MBPbIqV92AaeXatLxBI9gBaebbnrfifHhDYfgasaacH8akY=wiFfYdH8Gipec8Eeeu0xXdbba9frFj0=OqFfea0dXdd9vqai=hGuQ8kuc9pgc9s8qqaq=dirpe0xb9q8qiLsFr0=vr0=vr0dc8meaabaqaciGacaGaaeqabaqabeGadaaakqaabeqaaiabdcfaqjabcIcaOiabdIfayjabcMcaPiabg2da9iabdAgaMjabcIcaOiabdIfaynaaBaaaleaacqWGmbataeqaaOGaeiilaWIaemyrauKaemOBa4MaemizaqMaeiykaKIaeyypa0ZaaabCaeaacqWGMbGzcqGGOaakcqWGybawdaWgaaWcbaGaemitaWeabeaakiabcYcaSiabd6gaUjabcMcaPiabdsfaunaaBaaaleaacqWGUbGBcqGGSaalcqWGfbqrcqWGUbGBcqWGKbazaeqaaaqaaiabd6gaUjabgUcaRiabigdaXaqaaiabd2eanbqdcqGHris5aOGaaCzcaiaaxMaacqGGOaakcqaI3aWncqGGPaqkaeaacqWGLbqzdaWgaaWcbaGaemyAaKgabeaakiabcIcaOiabdMha5jabcYcaSiabdIfayjabcMcaPiabg2da9iabdwgaLnaaBaaaleaacqWGPbqAaeqaaOGaeiikaGIaemyEaKNaeiilaWIaemiwaG1aaSbaaSqaaiabdYeambqabaGccqGGSaalcqWGfbqrcqWGUbGBcqWGKbazcqGGPaqkcqGH9aqpdaaeWbqaaiabdwgaLnaaBaaaleaacqWGPbqAaeqaaOGaeiikaGIaemyEaKNaeiilaWIaemiwaG1aaSbaaSqaaiabdYeambqabaGccqGGSaalcqWGUbGBcqGGPaqkcqWGubavdaWgaaWcbaGaemOBa4MaeiilaWIaemyrauKaemOBa4MaemizaqgabeaaaeaacqWGUbGBcqGHRaWkcqaIXaqmaeaacqWGnbqta0GaeyyeIuoaaaaa@890A@

**Proof: **The proof is strictly analogous to the proof of theorem 1.

The above theorems have shown that *t*_*i*, *j*_(*X*) and *e*_*i*_(*y*, *X*) can each be calculated in *O*(*M*) memory and *O*(*LMT*_*max*_) time. As there are *T *transition parameters and *E *emission parameters to be calculated in each Baum-Welch iteration, and as these *T *+ *E *values can be calculated independently, the time and memory requirements for each iteration and a set of training sequences whose sum of sequence lengths is *L *using our new algorithm are

• *O*(*M*) memory and *O*(*LMT*_*max *_(*T *+ *E*)) time, if all parameter estimates are calculated consecutively

• *O*(*M*(*T *+ *E*)) memory and *O*(*LMT*_*max*_) time, if all parameter estimates are calculated in parallel

• more generally, *O*(*Mc*) memory and *O*(*LMT*_*max *_(*T *+ *E *- *c*)) time for any *c *∈ {1,..., (*T *+ *E*)}, if *c *of *T *+ *E *parameters are to be calculated in parallel

Note that the calculation of *P*(*X*) is a by-product of each *t*_*i*, *j*_(*X*) and each *e*_*i*_(*y*, *X*) calculation, see Equations 4 and 7, and that *T *is equal to the number of free transition parameters in the HMM which is usually smaller than the number of transitions probabilities. Likewise, *E *is the number of free emission parameters in the HMM which may differ from the number of emission probabilities when the probabilities are parametrised.

## Discussion and Conclusion

We propose the first linear-memory algorithm for Baum-Welch training. For a hidden Markov model with *M *states, *T *free transition and *E *free emission parameters, and an input sequence of length *L*, our new algorithm requires *O*(*M*) memory and *O*(*LMT*_*max *_(*T *+ *E*)) time for one Baum-Welch iteration as opposed to *O*(log(*L*)*M*) memory and *O*(log(*L*)*LMT*_*max *_+ *L*(*T *+ *E*)) time using the checkpointing algorithm [11-13], where *T*_*max *_is the maximum number of states that any state is connected to. Our algorithm can be generalised to pair-HMMs and, more generally, n-HMMs that analyse n input sequences at a time in a straightforward way. In the n-HMM case, our algorithm reduces the memory and time requirements from *O*(log(*L*)*L*^*n*-1 ^*M*) memory and *O*(log(*L*)*L*^*n *^*MT*_*max *_+ *L*^*n*^(*T + E*)) time to *O*(*L*^*n*-1 ^*M*) memory and *O*(*L*^*n *^*MT*_*max *_(*T *+ *E*))) time. An added advantage of our new algorithm is that a reduced time requirement can be traded for an increased memory requirement and *vice versa*, such that for any *c *∈ {1,..., (*T *+ *E*)}, a time requirement of *L*^*n *^*MT*_*max *_*c *incurs a memory requirement of *L*^*n*-1 ^*M*(*T *+ *E *- *c*). For HMMs, our novel algorithm renders the memory requirement completely independent of the sequence length. Generally, for n-HMMs and all *T *+ *E *parameters being estimated consecutively, our novel algorithm reduces the memory requirement by a factor log(*L*) and the time requirement by a factor log(*L*)/(*T *+*E*) + 1/(*MT*_*max*_). For all hidden Markov models whose number of states does not depend on the length of the input sequence, this thus amounts to a significantly reduced memory requirement and – in cases where the number of free parameters and states of the model (i.e. *T *+ *E*) is smaller than the logarithm of sequence lengths – even to a reduced time requirement.

For example, for an HMM that is used to predict human genes, the training sequences have a mean length of at least 2.7·10^4 ^bp which is the average length of a human gene [14]. Using our new algorithm, the memory requirement for Baum-Welch training is reduced by a factor of about 28 ≈ e* In (2.7·10^4^) with respect to the most memory-sparse version of the checkpointing algorithm.

**Figure 1:**
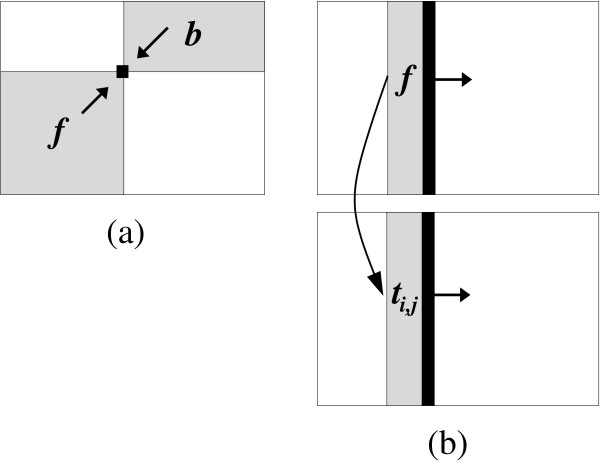
**Pictorial description of the new algorithm for pair-HMMs**. This figure shows a pictorial description of the differences between the forward-backward algorithm (a) and our new algorithm (b) for the Baum-Welch training of a pair-HMM. Each large rectangle corresponds to the projection of the three-dimensional dynamic programming matrix (spanned by the two input sequences *X *and *Y *and the states of the HMM) onto the sequence plane. (a) shows how the numerator in Equation 1 is calculated at the pair of sequence positions indicated by the black square using the standard forward and backward algorithm. (b) shows how our algorithm simultaneously calculates a strip of forward values *f*(*X*_*k*_, *Y*_*q*_, *m*) and a strip of *t*_*i*, *j*_(*X*_*k*_*Y*_*q*_, *m*) values at sequence position *k *in sequence *X *in order to estimate *t*_*i*, *j *_in Equation 1.

Our new algorithm makes use of the fact that the numerators and denominators of Equations 1 and 2 can be decomposed in a smart way that allows a very memory-sparse calculation. This calculation requires only one *uni*-directional scan along the sequence rather than one or more *bi*-directional scans, see Figure 1. This property gives our algorithm the added advantage that it is easier to implement as it does not require programming techniques like recursive functions or checkpoints.

Baum-Welch training is only guaranteed to converge to a *local *optimum. Other optimisation techniques have been developed in order to find better optima. One of the most successful methods is simulated annealing (SA) [1,15]. SA is essentially a Markov chain Monte Carlo (MCMC) in which the target distribution is sequentially changed such that the distribution gets eventually trapped in a local optimum. One can give proposal steps a higher probability as they are approaching locally better points. This can increase the performance of the MCMC method, especially in higher dimensional spaces [16]. One could base the candidate distribution on the expectations such that proposals are more likely to be made near the EM updates (calculated with our algorithm). There is no need to update all the parameters in one MCMC step, modifying a random subset of parameters yields also an irreducible chain. The last feature makes SA significantly faster than Baum-Welch updates as we need to calculate expectations only for a few parameters using SA. In that way, our algorithm could be used for highly efficient parameter training: using our algorithm to calculate the EM updates in only linear space and using SA instead of the Baum-Welch algorithm for fast parameter space exploration.

Typical biological sequence analyses these days often involve complicated hidden Markov models such as pair-HMMs or long input sequences and we hope that our novel algorithm will make Baum-Welch parameter training an appealing and practicable option.

Other commonly employed methods in computer science and Bioinformatics are stochastic context free grammars (SCFGs) which need *O*(*L*^2 ^*M*) memory to analyse an input sequence of length *L *with a grammar having *M *non-terminal symbols [1]. For a special type of SCFGs, known as covariance models in Bioinformatics, *M *is comparable to *L*, hence the memory requirement is *O*(*L*^3^). This has recently been reduced to *O*(*L*^2 ^log(*L*)) using a divide-and-conquer technique [17], which is the SCFG analogue of the Hirschberg algorithm for HMMs [9]. However, as the states of SCFGs can generally impose long-range correlations between any pair of sequence positions, it seems that our algorithm cannot be applied to SCFGs in the general case.

## Authors' contributions

The algorithm is the result of a brainstorming session of the authors on the Genome campus bus back to Cambridge city centre on the evening of the 17th February 2005. Both authors contributed equally.
